# Knowledge, skills and beetles: respecting the privacy of private experiences in medical education

**DOI:** 10.1007/s40037-020-00565-5

**Published:** 2020-02-05

**Authors:** Mario Veen, John Skelton, Anne de la Croix

**Affiliations:** 1 Department of General Practice, Erasmus University Medical Center, Rotterdam, The Netherlands; 2 Institute of Clinical Sciences, University of Birmingham, Birmingham, UK; 3 Educational Sciences, Faculty of Behaviour and Movement Sciences, VU University, Amsterdam, The Netherlands; 4 Research in Education, Amsterdam UMC, VUmc School of Medical Sciences, Amsterdam, The Netherlands

**Keywords:** Communication, Empathy, Professionalism, Competency based education, Philosophy, Assessment

## Abstract

In medical education, we assess knowledge, skills, and a third category usually called values or attitudes. While knowledge and skills can be assessed, this third category consists of ‘beetles’, after the philosopher Wittgenstein’s beetle-in-a-box analogy. The analogy demonstrates that private experiences such as pain and hunger are inaccessible to the public, and that we cannot know whether we all experience them in the same way. In this paper, we claim that unlike knowledge and skills, private experiences of medical learners cannot be objectively measured, assessed, or directly accessed in any way. If we try to do this anyway, we risk reducing them to knowledge and skills—thereby making curriculum design choices based on what can be measured rather than what is valuable education, and rewarding zombie-like student behaviour rather than authentic development. We conclude that we should no longer use the model of representation to assess attitudes, emotions, empathy, and other beetles. This amounts to, first of all, shutting the door on objective assessment and investing in professional subjective assessment. Second, changing the way we define ‘fuzzy concepts’ in medical education, and stimulating conversations about ambiguous terms. Third, we should reframe the way we think of competences and realize only part of professional development lies within our control. Most importantly, we should stop attempting to measure the unmeasurable, as it might have negative consequences.

## Introduction

*Suppose every medical student had a box with something in it: we call it ‘empathy’. No one can look into anyone else’s box, and all students say they know what empathy is only by looking at their own experience of empathy.—Here it would be quite possible for everyone to have something different in their box. One might even imagine such a thing constantly changing.*

We could replace the word ‘empathy’ in this quote by ‘integrity’, ‘reflection’, or ‘professional identity formation’—these are terms that have found their way into our common medical educational language yet are intensely personal and private for learners. We will call them ‘beetles’, after the thought experiment by Ludwig Wittgenstein:

*Suppose everyone had a box with something in it: we call it a ‘beetle’. No one can look into anyone else’s box, and everyone says he knows what a beetle is only by looking at his beetle.—Here it would be quite possible for everyone to have something different in their box. One might even imagine such a thing constantly changing *[1]*.*

In the beetle-in-a-box analogy, the beetle stands for private, personal experiences. We can never know if other people experience empathy or pain in the same way, because we can never take our own inner experiences out of the ‘box’, and compare them.

We shall follow this fundamental idea through, before considering ways to deal with beetles in medical education. The meanings of words that refer to private experiences (‘pain’, ‘sadness’) are not to be found in a mental image. The less we can point to something (‘THIS is what I mean when I say ‘chair’’), the more complex it is to define. It is difficult to find words for our *own* private experiences, but it is impossible to do so for someone else’s inner world. However, medical education has recently shown more interest in the personal and emotional side of medicine, and has thereby introduced such fuzzy concepts [2] as empathy, professional identity development, and reflection. These are beetles in the individual’s box, carried through the student years and the subsequent career.

For our purposes, the core of the beetle problem is that while we know private experiences exist and matter for developing into a competent healthcare professional, we cannot observe, measure, or share them, because there is no external referent that both parties can point to. This means it is hard to incorporate beetles into the medical curriculum, in which assessment requires demonstration and observation, where the assumption is that what we observe is a representation of the ‘real thing’. A skill can be demonstrated, knowledge can be demonstrated. Beetles cannot.

In medical education, however, we often act as if medical students are able to open up their boxes and produce the contents for assessment purposes. Educators, in turn, are expected to assess medical students’ levels of empathy [3] or the quality of their reflection [4]. To this end, medical trainees are increasingly asked to ‘share’ private experiences in the medical curriculum. But what if this is fundamentally impossible?

We do not know how to deal with beetles-in-boxes. At the same time, we acknowledge that at this point in the young science of competency-based medical education we are still ‘in the midst of a transition in which we cannot remain standing ’ [5]. So let us first look at the way in which phenomena beyond knowledge and skills entered medical schools.

## How beetles entered medical curricula

There is no exact moment when beetles, different animals from knowledge and skills, entered formal curricula. We hypothesize that two main developments played a role.

First, the focus on the safety and care of the patient. A focus on the lay rather than the professional has been with us for a century, in Dewey for general education [6], and in Osler for medicine *passim* [7]. From the 1970s [8] onwards, there have been increasing calls for humanized medicine, with its emphasis on the patient as well as the disease [9]. A major impetus in the UK was the response to the tragedy at Bristol Royal Infirmary, where there were serious problems in paediatric cardiac surgery. Kennedy spoke of the need to ‘broaden the notion of competence’, to include such things as communication, team-work and the like [10]. Human factors and patient-centredness became important aspects of healthcare [11, 12].

Second, the movement toward outcome-based education, and consequently the burgeoning use of the OSCE approach offered itself explicitly as an objective way of assessing competence [13–17]. Insights from educational sciences were used as an inspiration and ‘a detailed analysis of the characteristics and qualifications of the modern physician was deemed necessary, in terms of skills, personality traits, social and economic problems, and responsibility as a citizen’ [13]. Taking guidance from ‘functions required for the practice of medicine in a specified setting’, doors opened for new and previously unexplored themes in medical education. The famous CanMeds roles can be seen as an offshoot of this development, as they are in part a response to changes that patients and society required from doctors. Among the CanMeds roles we find ‘professional’, ‘communicator’, and ‘collaborator’ [18].

In grappling with CanMeds roles, many strange guests entered the formal curriculum: integrity, professionalism, empathy, reflection, a caring attitude, and so on. These new concepts had to comply with some of the demands of their new home: beetles were immediately clearly defined to be measurable and assessable—as is customary in systems under scrutiny of accreditation bodies. This demand for conceptual clarity was quickly satisfied, transforming a beetle-like empathy into an ‘achievement’ or ‘performance’, a set of behaviours that can be taught and demonstrated, and therefore assessed and measured. However, the attempt to find a uniform definition hinges on the assumption that when we are using the same word, we mean the same thing.

Beetles were added to the curriculum to make medicine more human, which is an endeavour we applaud. But the cost has been to reduce them to knowledge and behaviours. When empathy becomes a skill or a performance, it ceases to be empathy. The moment reflection becomes a tick-box exercise, it ceases to be authentic reflection [19]. This paper explores the problems we encounter in dealing with beetles in medical education, and how to move forward. So first, we will focus on the complexity of beetles.

## The complexity of beetles

Wittgenstein’s beetle-in-a-box thought experiment deals principally with sensations, but the argument can be extended, as we do in this paper. Wittgenstein deals in particular with the question of *pain*. Is my experience of pain the same as yours? Are we talking about the same thing? There are two points to be made here about beetles in medical education.

First, words change their meaning, and the community uses them in ways which reflect and reinforce these changes—or the changes die out. Thus, English ‘gay’ seems unusable now in the sense of ‘light-hearted’. ‘Square’ can no longer mean ‘unfashionable’, as it did in the 1950s. Meaning is determined by use. Studying changing meanings of words in medical education can teach us a lot, as can be seen in a study about perceptions of the ‘competent’ doctor [20]. Kripke suggests that in using a sign (i.e. a word), what matters is that the ‘community’ (in our case, the medical education community) agree that I have used it successfully [21]. Medical education consists of different cultural and institutional communities, and concepts that are being used in medical education have often travelled from other scientific communities (medical science, psychology, humanities, social & educational sciences). In their travels across individuals, communities, and scientific disciplines, words take on meanings and lose others [22]. This might explain why a concept such as ‘emotion’ can take on very different meanings [23].

Second, concepts associated with value are in principle never well-defined. A plus sign in mathematics is clearly defined: that is, the meaning assigned by the community to the activity designated by the symbol is well-defined. This is broadly true of a great many medical terms, for parts of the body and names of diseases, for example. There is no such agreement—there can be no such agreement—about a term like ‘empathy’, or ‘respect’ (for colleagues or patients, for example). And beyond that, it seems clear that ‘empathy’ does not consist of a list of things to do. An empathic person may behave in specific ways (cocks their head, listens carefully, says ‘that must be very hard for you’), but failure to do so could mean many things. We might quickly infer a lack of empathy, yet it could mean that this person does not know how to demonstrate the value or attitude of ‘empathy’ in a way that the community can understand, as might be the case in many young undergraduates. Another option is that this person has a different background to us, the perceivers, and we might not interpret their behaviour as displaying a certain emotion [24]. Equally, a cynical student may imitate empathic behaviour in order to pass exams.

**Fig. 1:**
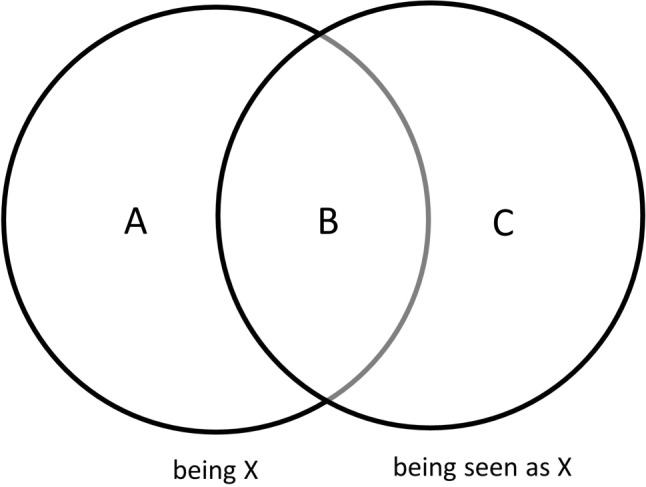
Venn diagram depicting what we can and cannot observe about students

There is no causal relationship between the ‘real’ beetle, and the set of behaviours and markers that might be seen as the manifestation of that beetle. Also, being seen as demonstrating a value or attitude is not evidence of actually possessing it. This means that sometimes, for example empathic students are seen as empathic (Fig. 1, Area B), sometimes students who do not actually experience empathy are indeed seen as empathic (Area C), and sometimes students who are not seen as empathic, actually are (Area A). The catch is that while we know that the two circles probably overlap somewhere, we are unable to tell which students fall into which area.

## Assessing beetles—an impossible venture

The real difficulty with beetles in medical education comes with testing. It is easy to teach and test aspects of knowledge (‘knowing’) and skills (‘doing’). The student learns, say, about first-line drugs for diabetes, and can be given an MCQ about them. Or they practice taking blood pressure and are tested on how accurately they can do it under exam conditions. Abstract concepts (beetles), however, are typically linked to values, and in principle not susceptible to precise definition. There is in that sense no exact way of telling a ‘right’ answer from a ‘wrong’ answer. But equally, one cannot assess whether a doctor has good values by asking them if it is okay to operate under the influence of alcohol. They are likely to know what answer is required.

The risk is that, for testing purposes, a beetle is defined in terms of what students must know or do. It is reformulated as a behaviour, and indicators are specified so it becomes measurable. But when the beetle is defined and tested only in terms of performance, what we have elsewhere called the Zombie problem [19] arises in two ways. First, as outside observers, we cannot say if the behaviour is authentic or an act, such as an actor might give. Second, in a system that views behaviour in this way, students are rewarded for just the behaviour, and there is no extrinsic motivation to actually engage in meaningful beetle education. (As has been pointed out [25], this is also a common theological argument. If wickedness—‘poor values’—were always punished, the universe would lose its moral purpose: we would all be cynically good).

So, for example, we want students to reflect. Sadly, we can easily posit a ‘reflective zombie’ [19], a student showing all the outer traits of a reflective person without having actually reflected. A student’s ability to write a reflective essay in which knowledge of reflection models has been applied does not guarantee actual reflection. It just means they are good at writing a reflective essay: there is a box, a space in which reflection could take place, but we do not know if there is a beetle in there, or if it contains anything at all.

The way we traditionally assess beetles is with the model of *representation*. In this model, language and action are seen as a representation of what is actually going on in people’s inner lives [26]. We recognize that we cannot look directly inside students’ heads and hearts. But what we *can* observe, the logic goes, is their behaviour: if a student says ‘well that must be hard for you’ to a patient in the right tone of voice at the appropriate moment in the history taking part of the medical interview, this observable behaviour must be indicative of a feeling of empathy ‘inside’ the student. We expect educators to interpret what a student does and says, and we expect them to translate this to a judgment of the student’s beetle—reflection, empathy, professional behaviour, attitude, or whatever the animal is called. This representational model has been criticized [1, 27, 28] but is still the dominant model in medical education. We believe it is time to leave this model behind, as we can never know what public behaviour represents, or if it represents anything at all. We can agree on the behavioural and physical markers of what we think a beetle in a box looks like, and we can ask people to self-report a score of their own beetle—but that does not tell us anything about the beetle itself.

## The way forward

We have outlined the beetle-in-a-box thought experiment, the representational approach to abstract concepts in medical education, and the consequent difficulties in attempting to measure beetles. If we accept that beetles are private, then what does this mean for the future of beetles in medical education? Is it appropriate to have competencies that are impossible to assess objectively? We will explore implications for assessment, definition, and competency-based education.

## Assessment: respect the privacy of the beetle

Given the limits to what is possible to know, measure, and assess, we challenge the reflex question that often occurs when introducing a concept in the curriculum: how will we assess this? We suggest educators ask themselves three preceding questions when dealing with educational developments:

1. ‘Do we want to assess this, and, if so, why?’ Does the intended assessment truly contribute to competency development? This opens up the possibility that there may be parts of the curriculum which are important, but not beneficial or even harmful to assess.
2. ‘Is it possible to assess this?’ If something cannot be objectively measured, and we apply a form of assessment anyway, then we need to think about what is *actually *being assessed.
3. ‘Which aspect of this phenomenon do we assess, and how?’ Here the assessment method should fit what is being assessed.

In answering this last question, we think it is helpful to consider subjective assessment.

Objective assessment of beetles is fundamentally impossible, because it requires an external standard. Standardization is not always possible: extrapolating, for example, how a doctor performs in a real-world environment from performance in a standardized test is unwise [29, 30], because: ‘The real world is non-standardized and haphazard, and, more importantly, any attempt at standardization will only trivialize the assessment’ [31].

As human beings, we form opinions and judgments of each other all the time. Subjective assessment means that the assessor’s experience is a necessary part of the equation [30]. An educator might form an idea of whether a student comes across as empathic by being aware how their own beetle ‘resonates’ in the conversation, using themselves as an instrument. Or we might look at patient feedback and their experience of empathy in a (mock) consultation. We are proposing a phenomenological approach to assessment, which means that the *experience *of the assessor, rather than an objective assessment, is the criterion. This kind of assessment can be very useful, as long as no pretences are made about the beetle (e.g., ‘this person cannot reflect’, or ‘this person is not empathic’). It also means that we have to accept that different assessors can have different assessments of the same person. This makes other forms of assessment more logical, such as programmatic assessment or holistic assessment [30].

This type of assessment requires teacher training, as the assessment ‘instrument’ is the teacher’s experience. This kind of training would focus on being aware of and reflecting on one’s limits as an assessor, and being aware of always being biased. High-quality educators will form high-quality judgments, as true professionals will be reflective and aware of their own subjectivity and potential bias.

## Definitions: beetle-shaped holes

The contemporary focus on the whole person of the future doctor, including their emotions [30], is welcome. However, any attempt to reach precise definitions is misguided. Operationalizing these definitions risks causing complex concepts to lose their essence. Professionalism, for example, might become a tick-box list of behaviours.

Rather than seeing beetles as something to be defined, we might see them as fuzzy concepts [2] that travel between communities, take on certain meanings, lose others, and leave room for individual interpretation. ‘While groping to define, provisionally and partly, what a particular concept may mean, we gain insight into what it can do. It is in the groping that the valuable work lies. […] Even those concepts that are tenuously established [are valuable] primarily because of their potential intersubjectivity. Not because they mean the same thing for everyone, but because they don’t’ [22].

Just as students of sociology, say, have to work with abstract concepts such as ‘capitalism’ or ‘anomie’ or ‘ascribed status’, so medical students will need to learn to handle terms like ‘patient-centredness’, ‘teamwork’, and ‘reflection’. The only reason anyone ever thought this was hard is because medical education is perceived as involving science, and science is about facts. The discussion of difficult concepts is, however, routine in social sciences and humanities. The aim is to deepen students’ understanding of the concepts (after all, not many medical students are articulate about ‘empathy’ on Day 1), and by extension help them to mount and sustain a rational argument. The tradition here—it is to some extent the tradition of learning itself—goes back, to Plato’s Socratic Dialogues [32], which are absolutely centrally concerned with the exploration of the abstract, and the development of arguments. After all, one might argue: if we do not want our doctors to be capable of clear thought, why on earth not?

This is not easy, as in the medical education system the aim is still to sort out what is ‘correct’ from what is ‘incorrect’, to use evidence to decide on the best course of action, and so on. In this tradition, every concept is well-defined, and if it is not, it is not a scientific concept and therefore not worth bothering with, at least professionally. This means educational developers, teachers, and students need to learn how to deal with ambiguity. Social science and the humanities have a lot to offer medical education in this respect.

## Competency frameworks: acknowledge the limits

The central conundrum is simply stated. If the condition for inclusion in the medical curriculum is that something is objectively measurable, then indeed beetles have no place in the curriculum, and we should focus only on knowledge and skills. Yet we know that there are things (integrity, values, self-awareness, to name but a few) which matter profoundly but which cannot be clearly defined, let alone assessed.

Competency frameworks set out aspects of knowledge and skills which can be measured. The act of measurement under examination conditions, however, and we should be clear about this, is a measurement of whether a doctor can *perform to order*. But a surgeon who can undertake a procedure when she/he is concentrating (their future is at stake) may nevertheless not be bothered to deploy the same level of care and concentration faced with an actual patient, at the end of a long shift, when they want to get home. Similarly, most students can perform empathy in an OSCE, face to face with a standardized patient: but this does not make them empathic.

Assessment of beetles, or rather, assessments of performances that we associate with beetles, requires well-trained people if it is to be attempted at all. Individuals with integrity, experience, self-awareness, conscious of the risk of unconscious bias and so on, and perhaps individuals with access to students on the wards, interacting in a real clinical environment. And as far as students are concerned, learning what kind of ‘animals’ beetles are, and exploring the beetles they personally carry with them, might require space for students to reflect, explore and talk to peers and mentors.

Assessment and measurement should not be goals in themselves, but serve a higher purpose: training doctors who can meet all the CanMeds roles. Assessments can have educational value—people learn from being assessed, learn from feedback, and from mapping their progress. But in many cases, assessment can be counterproductive and guide students towards superficial learning behaviour.

The essence of the beetle-in-a-box thought experiment is that the beetle ‘itself’ cannot play any very well-defined role in public language. Your concept of ‘honesty’ may be different from mine, even if we suspect we know roughly what we are talking about. We should, therefore, respect the privacy of the beetle. One definition of privacy [33] is ‘the feature which leaves each person’s experiences and thoughts as known immediately to that person’ [34]. The beetle analogy shows that we as educators have no immediate access to those inner states. Only the students themselves do.

## Conclusion

Next to knowledge and skills, beetles-in-boxes play an important part in medical education. Beetles are private experiences that are inaccessible to the outer world, but are an important part of the way individuals experience the world and thus the way in which they learn and develop. The problem with beetles is that we treat them as skills and ask students to demonstrate them (which is impossible), we try to measure them (which is impossible), and thereby we stimulate superficial ‘zombie’ learning behaviour. We call for medical educators to respect the privacy of private experiences by not trying to see, measure, or assess them. Rather than doing so, we propose to shift the focus to the person holding the beetle, the medical student. Beetles cannot be assessed, but the experience of the assessor can be a valuable resource. Beetles cannot be defined, but concepts that allow for diversity in meaning can be food for reflection, development, and discussion. Together with knowledge and skills, beetles-in-boxes are an essential part of competency frameworks—as long as we accept that no one can look in anyone else’s box.

## References

[1] Wittgenstein L (1953). Philosophical investigations.

[2] Haack S (1997). Deviant logic, fuzzy logic : beyond the formalism.

[3] Sulzer SH, Feinstein NW, Wendland CL (2016). Assessing empathy development in medical education: a systematic review. Med Educ.

[4] Aukes LC, Geertsma J, Cohen-Schotanus J, Zwierstra RP, Slaets JP (2007). The development of a scale to measure personal reflection in medical practice and education. Med Teach.

[5] Rilke RM, Herter Norton MD (1934). Letters to a young poet.

[6] Dewey J, Nagel E (1991). Logic, the theory of inquiry.

[7] Osler BMW (1999). A life in medicine.

[8] Ferreira-Padilla G, Ferrández-Antón T, Baleriola-Júlvez J, Braš M, Đorđević V (2015). Communication skills in medicine: where do we come from and where are we going?. Croat Med J.

[9] General Medical C, Education C (1993). Tomorrow’s doctors : recommendations on undergraduate medical education.

[10] Kennedy I (2001). Final report: Bristol royal infirmary inquiry.

[11] Kohn LT, Corrigan JM, Donaldson MS, Institute of Medicine Committee on Quality of Health Care in A (2000). To err is human: building a safer health system.

[12] Balint E (1969). The possibilities of patient-centered medicine. J R Coll Gen Pract.

[13] Custers E, Cate OT (2018). The history of medical education in Europe and the United States, with respect to time and proficiency. Acad Med.

[14] Harden RM (1999). AMEE Guide No. 14: outcome-based education: Part 1-An introduction to outcome-based education. Med Teach.

[15] Ten Cate O (2017). Competency-based postgraduate medical education: past, present and future. GMS J Med Educ.

[16] Hodge S (2007). The origins of competency-based training. Aust J Adult Learn.

[17] Morcke AM, Dornan T, Eika B (2013). Outcome (competency) based education: an exploration of its origins, theoretical basis, and empirical evidence. Adv Health Sci Educ Theory Pract.

[18] Frank JR, Danoff D (2007). The CanMEDS initiative: implementing an outcomes-based framework of physician competencies. Med Teach.

[19] de la Croix A, Veen M (2018). The reflective zombie: problematizing the conceptual framework of reflection in medical education. Perspect Med Educ.

[20] Whitehead CR, Austin Z, Hodges BD (2013). Continuing the competency debate: reflections on definitions and discourses. Adv Health Sci Educ.

[21] Kripke SA (1982). Wittgenstein on rules and private language: an elementary exposition.

[22] Bal M (2012). Travelling concepts in the humanities: a rough guide.

[23] McNaughton N (2013). Discourse(s) of emotion within medical education: the ever-present absence. Med Educ.

[24] Engelmann JB, Pogosyan M (2013). Emotion perception across cultures: the role of cognitive mechanisms. Front Psychol.

[25] Skelton J (2008). Language and clinical communication: this bright Babylon.

[26] Potter J, Edwards D (1999). Social representations and discursive psychology: from cognition to action. Cult Psychol.

[27] Austin J (1975). How to do things with words.

[28] Potter J (2012). Representing reality: discourse, rhetoric and social construction.

[29] Norman GR, Van der Vleuten CP, De Graaff E (1991). Pitfalls in the pursuit of objectivity: issues of validity, efficiency and acceptability. Med Educ.

[30] Schuwirth L, Ash J (2013). Assessing tomorrow’s learners: In competency-based education only a radically different holistic method of assessment will work. Six things we could forget. Med Teach.

[31] van der Vleuten CP, Schuwirth LW, Driessen EW (2012). A model for programmatic assessment fit for purpose. Med Teach.

[32] Plato JB (1961). The four Socratic dialogues.

[33] Feldges T, Gray JNW, Burwood S (2014). Subjectivity and the social world a collection of essays around issues relating to the subject, the body and others.

[34] Hannay A, Honderich T (2005). Privacy. The Oxford companion to philosophy.

